# Atherogenic Dyslipidemia and Cardiovascular Risk Factors in Obese Children

**DOI:** 10.1155/2015/912047

**Published:** 2015-01-12

**Authors:** Ebe D'Adamo, Ornella Guardamagna, Francesco Chiarelli, Andrea Bartuli, Daniela Liccardo, Federica Ferrari, Valerio Nobili

**Affiliations:** ^1^Unit of Pediatrics, Hospital of Cremona, Largo Priori 1, 26100 Cremona, Italy; ^2^Department of Health Science and Pediatrics, University of Turin, Piazza Polonia 94, 10126 Turin, Italy; ^3^Department of Pediatrics, University of Chieti, Via Dei Vestini 5, 66013 Chieti, Italy; ^4^Rare and Genetic Diseases Unit, Bambino Gesù Children's Hospital, Piazza S. Onofrio 4, 00165 Rome, Italy; ^5^Hepatometabolic Diseases Unit, Bambino Gesù Hospital, Piazza S. Onofrio 4, 00135 Rome, Italy; ^6^Pediatric Department, Pediatric Gastroenterology and Liver Unit, Umberto I Hospital, Sapienza University of Rome, Viale Regina Elena 324, 00161 Rome, Italy

## Abstract

Childhood obesity when associated with serum lipoprotein changes triggers atherosclerosis. Evidences suggest that the atherosclerotic process begins in childhood and that the extent of early atherosclerosis of the aorta and coronary arteries can be associated with lipoprotein levels and obesity. Furthermore, many studies in childhood demonstrate an important relationship between parameters of insulin sensitivity, body fat distribution, and the development of lipid abnormalities. This review focuses on the most recent findings on the relationship between obesity, dyslipidemia, and cardiovascular risk in children.

## 1. Introduction

Childhood obesity represents one of the most important public health issues, due to its associated metabolic and cardiovascular comorbidities [1]. In spite of public health policies to prevent pediatric obesity, its prevalence has not shown a decrease [2].

The prevalence of childhood obesity remains high not only in the United States [3]. In the World Health Organization (WHO) European Region the estimated prevalence of obesity is around 20% [4]. According to the findings of the IDEFICS (identification and prevention of dietary and lifestyle induced health effects in children and infants) carried out in a cohort of 18 745 very young children (2.0–9.9 years) from eight European countries, the prevalence of overweight/obesity ranges from more than 40% in southern Europe to less than 10% in northern Europe. Furthermore, it was higher in girls and in populations with lower education and income levels [4]. In accordance with the latest, in Italy around 30000 to 50000 children aged 8-9 years are obese, with higher prevalence in the south areas [3].

During the last decade, the prevalence of atherogenic dyslipidemia is increasing in obese children and adolescents [4, 5]. Data from the Third National Health and Nutritional Examination Survey (NHANES III) indicate that 25% of adolescents are characterized by high triglycerides (TG) levels and 40% by low high density lipoprotein (HDL) cholesterol levels [6]. Investigators from Bogalusa Heart Study reported that overweight schoolchildren were 2.4 to 7.1 times more likely to have elevated total cholesterol (TC), low density lipoprotein (LDL) cholesterol, and TG than their lean counterparts [7, 8].

The atherogenic dyslipidemia is characterized by hypertriglyceridemia, very-low-density lipoprotein (VLDL) increase, small dense LDL (sdLDL) particles, and HDL cholesterol reduced levels [9] thus showing components of the metabolic syndrome (MetS) [1, 9], a relevant cause of cardiovascular disease [5, 9].

Among different features that characterize MetS the lipoprotein profile plays a basic role [1]. The ratios of TG/HDL cholesterol and low density lipoprotein (LDL)/HDL cholesterol are well-established predictors of cardiovascular disease [10]. Furthermore, the ratios of TG/HDL and apolipoprotein B (ApoB)/apolipoprotein A-1 (ApoA-1) have been shown to be good predictors of MetS and cardiovascular disease [10, 11].

Evidence exists suggesting that the atherosclerotic process begins in childhood [12, 13] and the extent of early atherosclerosis of the aorta and coronary arteries is directly associated with levels of lipoproteins, blood pressure, and obesity in childhood and adolescence [12, 13]. Notably, Giannini et al. [12] have demonstrated an impaired endothelial dysfunction already in prepubertal age group and a direct association between early signs of atherosclerosis, obesity, and impaired insulin sensitivity.

Moreover, overweight adolescents who remained overweight in adulthood had, respectively, 2.4, 3, and 8 times greater prevalence of abnormal LDL cholesterol, TG, and HDL cholesterol concentrations than those who remained lean [14].

Although the pathogenetic mechanism of dyslipidemia is multifactorial and still debated, visceral obesity and insulin resistance represent one of the most important markers of its progression.

In the present review we illustrate the most recent findings concerning the impact of insulin resistance and visceral distribution on dyslipidemia in obese children and adolescents. Furthermore, we describe the relationship between atherogenic dyslipidemia and cardiovascular disease in pediatric age and the current knowledge regarding methods to study the lipid profile.

## 2. Lipid Profile, Insulin Resistance, and Obesity in Children

### 2.1. Dyslipidemia and Insulin Resistance

Obese children and adolescents have been observed to have a more unfavourable lipid profile than children and adolescents with normal body weight [4, 15]. The Lipid Research Clinics Population Studies Data Book [7] and the Bogalusa Heart Study [8] have shown that obese adolescents had an abnormal “atherogenic” lipid profile consisting of elevated LDL cholesterol and TG and low HDL cholesterol compared to normal-weight children [4, 8].

Although the pathophysiology underlying the development of dyslipidemia in obese children is multifactorial and not yet completely defined, insulin resistance has been hypothesized to play a major role in the relationship between dyslipidemia and obesity [4, 16]. Several studies have demonstrated that dyslipidemia is associated with hyperinsulinemia/insulin resistance [17, 18] and type 2 diabetes [19]. By evaluating 82 obese adolescents and 40 lean subjects, Steinberger et al. [18] observed that the degree of insulin resistance explained a significant proportion of the variance in the levels of TG, LDL cholesterol, and HDL cholesterol. Furthermore, Stan et al. [20] have estimated a prevalence of sdLDL particles of 10% in children showing insulin resistance compared to 1% in those without insulin resistance.

It has to be acknowledged that some studies defined insulin resistance on the basis of fasting insulin values or insulin sensitivity indices. Interestingly, Burns et al. [16] have evaluated the prevalence of atherogenic lipoprotein phenotype in 226 black and white obese children and adolescents aged 8–18 years and the relationship between lipoproteins and insulin sensitivity, as assessed by hyperinsulinemic-euglycemic clamp. The authors observed that children with greater degree of insulin resistance had a higher risk to develop atherogenic dyslipidemia, characterized by higher concentrations of sdLDL, small HDL, and large VLDL than those with a moderate insulin resistance status [16].

To strengthen the relationship between lipid profile and impaired insulin sensitivity, recent evidences [17] have demonstrated a link between the serum lipoprotein ratio and insulin resistance in adults as well as in children. In the study by Giannini et al. [17] the TG/HDL ratio was significantly associated with insulin resistance in a large cohort of obese youths. Interestingly de Giorgis et al. [21] have been able to confirm the association between TG/HDL ratio and early signs of vascular damage in obese prepubertal children.

The precise mechanisms by which insulin resistance leads to the development of atherogenic dyslipidemia remain unknown; however several pathways have been postulated [7, 22]. Insulin is a regulator of adipocyte functions and adipocytes show high response to insulin, which promotes the differentiation of preadipocytes to adipocytes and regulates lipogenesis and lipolysis [22]. In the insulin resistance status fatty acid (FA) esterification and an increased lipolysis occurring in adipocytes are defective. This condition is possibly due to the reduced insulin-mediated suppression of hormone-sensitive lipase (HSL) leading to an increased process of unesterified FA mobilization from the visceral fat depot [22, 23]. In addition, there is a decreased clearance of TG-rich lipoproteins in the circulation due to decreased lipoprotein lipase activity [22]. Thus, the FA flux to the liver is accelerated and affects adversely the hepatic insulin sensitivity, leading to increased production of TG and VLDL secretion.

Interestingly, Karpe et al. [24] have recently revised the concept that nonesterified fatty acids (NEFA) mobilization from visceral fat depots is accelerated in the insulin resistant state. By reviewing the most important studies in this field, the authors concluded that as adipose tissue mass expands, NEFA release per kilogram adipose tissue is downregulated, not increased, leading to the normalization of plasma NEFA levels in obese subjects. More studies are needed to clarify the relationships between obesity and FA kinetics.

### 2.2. Dyslipidemia and Body Fat Distribution

Obesity is the most important cause in the development of insulin resistance and several findings have shown that the critical determinant of insulin sensitivity and its related complications is not the degree of obesity “*per se*” but the distribution of fat partitioning [25]. By stratifying a multiethnic cohort of obese adolescents into tertiles based on the proportion of visceral and subcutaneous fat, Taksali et al. [25] observed significant increased TG levels, decreased HDL-cholesterol levels, and insulin sensitivity in the group with high proportion of visceral fat and low abdominal subcutaneous fat. Moreover, studies in children [26, 27] have demonstrated that the phenotype of subjects with high prevalence of small LDL particles was characterized by a greater degree of abdominal obesity. In the study by Burns et al. [16], visceral fat and insulin sensitivity, while independently or together, explained 26% of the variance in LDL size and 41% of the variance in HDL size. In addition, 12% of the variance in VLDL size has been explained by visceral fat.

### 2.3. Dyslipidemia and NAFLD

In spite of the demonstrated relationship between visceral fat, insulin resistance, and dyslipidemia, the ectopic fat deposition in the liver is emerging as one of the most important markers of metabolic abnormalities in children [28].

Pediatric nonalcoholic fatty liver disease (NAFLD) is becoming the most frequent chronic liver disease in obese children and adolescents. A growing body of evidence from epidemiologic studies in both adults and children has established NAFLD as an independent predictor for development of MetS, diabetes, and cardiovascular disease [29, 30]. Similarly to adults, children with NAFLD have a higher prevalence of atherosclerosis when compared to control subjects, as shown by increased carotid intima media thickness (cIMT) compared with matched controls [31, 32].

In the study by Schwimmer et al. [33], the authors demonstrated significantly higher TC, TG and lower HDL cholesterol levels in biopsy proven NAFLD children than controls. Significantly, among 49 obese adolescents with normal glucose tolerance, the presence of fatty liver, as assessed by fast magnetic resonance imaging, was associated with an increased concentration of large VLDL, sdLDL and decreased number of large HDL particles [26]. Interestingly, hepatic steatosis was found to predict the concentration of the large VLDL particles, independently of insulin sensitivity and visceral adiposity [26]. The same study group has recently compared the different influence of visceral fat and fatty liver in the development of atherogenic dyslipidemia in a multiethnic group of obese adolescents [27]. Liver fat accumulation has been shown to be an important predictor in large VLDL particle concentrations, while visceral fat was a significant predictor for large HDL and total small LDL concentrations [27]. It has to be noted that the role of insulin resistance in the development of dyslipidemia could change when related to the ethnic group. In fact, in the latest study [27] African American children, despite being hyperinsulinemic compared with Whites and Hispanics, have shown a more favourable lipid profile than the other groups. Although the factors responsible for these interracial differences are still unknown, along with genetic factors [27], fat distribution strongly contributes to the different lipoprotein profile observed among ethnicities [16, 28].

Several studies [16, 28] have attributed the lower concentrations of TG to the lower accumulation of visceral adipose tissue, typically seen in African Americans. Interestingly, in the study by D'Adamo et al. [27], at similar concentrations of visceral fat, African Americans obese adolescents had lower liver fat contents than Whites and Hispanics. Furthermore, liver fat accumulation, independent of visceral fat and insulin resistance, has been showed to be an important predictor in large VLDL particle concentrations among the three ethnic groups [27].

In accordance with the latest, Nobili et al. [34] have demonstrated that, in children with NAFLD, the severity of liver injury, as assessed by liver biopsy, is associated with markers of atherogenic profile. Notably, in children with NAFLD the relationship between liver damage and the atherogenic profile was independent of obesity, IR, and the presence of MetS [34].

Thus, although insulin resistance plays an important role in the development of atherogenic dyslipidemia in obese children, the abdominal fat deposition is emerging as an important link between insulin resistance and lipid alterations in obese children and adolescents.

## 3. Atherogenic Dyslipidemia and Cardiovascular Disease

Although atherosclerotic cardiovascular disease seems to be rare in the paediatric age group, the atherosclerotic process and the risk factors associated with its development begin in childhood [35].

The coronary atherosclerosis is a complex trait that recognizes multiple risk factor variables. In the Bogalusa Heart Study the prevalence of fatty streaks was 50% during childhood and 85% during young adulthood and the prevalence of fibrous plaques increased from 8% in childhood to 70% in young adulthood [36].

Longitudinal cohort studies from childhood conducted in Bogalusa, Louisiana (USA) [37], in Muscatine, Iowa (USA) [38], and in Finnish youths [39] demonstrated that several risk factors are associated with coronary change and predict the endothelial damage. Family history of premature coronary heart disease in a young adult cohort has a well-known association with low physical activity and smoking, obesity, diabetes, high blood pressure, and abnormal lipid levels [39]. The same observations from long-term pediatric studies, now extending into middle age, illustrate the tracking of risk factors and their adverse effects on the cardiovascular system. Childhood LDL cholesterol levels are highly predictive of adult levels into middle age [40], as obesity or hypertension. Notably, the Pathologic Determinants of Atherosclerosis in Youth (PDAY) study [41] showed that high levels of total cholesterol and hypertension represent two important risk factors for the development of endothelial damage.

Lipoproteins represent important atherosclerotic determinants and changes in the main lipoprotein fractions characterize the MetS. These changes involve a cascade of events including increased TG and sdLDL levels and decreased HDL cholesterol (below optimal levels), despite normal LDL cholesterol levels [42]. The cornerstone of lipid biochemical phenotype, in terms of cardiovascular risk, is ascribed to the small, dense phenomenon that applies to all lipoprotein particles [26, 27].

The role of TG has been controversial for decades [23] and even if more recent epidemiologic studies demonstrate that plasma TG levels predict cardiovascular disease [43], their role is still questionable. Baseline TG levels have been shown to predict cardiovascular disease mortality among relatives in families with familial hypertriglyceridemia as well as relatives in families with familial combined hyperlipemia [43], addressing new prevention strategies in these subjects.

Increasing TG levels cause profound changes in the physicochemical composition of HDL, VLDL, and LDL particles, and the particle core, represented by cholesterol esters, is progressively depleted and replaced by TG. The remodeling of larger to smaller lipoprotein particles is promoted by lipoprotein lipase and hepatic lipase [44], active in hydrolyzing TG core and phospholipids of lipoprotein particles, and by cholesteryl ester transfer protein, which mediates TG enrichment of intermediate and large density lipoproteins LDL [44]. These mechanisms appear to be major contributors to the production of sdLDL and to the reduction of large buoyant HDL2 lipoproteins leading to the development of metabolic complications [42].

A variable degree of correlation between coronary cardiovascular events and LDL size has been observed to vary with age, gender, and ethnicity [16]. The prevalence of sdLDL pattern is less represented in youths than adults but increases in childhood when insulin resistance syndrome occurs. In adolescents visceral adipose tissue was found to be related to the sdLDL phenotype even when the percentage body fat was not [45] demonstrating this proatherogenic profile since young age, when obese subjects are considered. A variability of LDL peak size is observed between children and adults and varies by different authors. The highest variation (50%–57%) was observed in the Framingham Offspring Study [46], while Stan observed a 30% variability [20] and a similar result was observed in Japanese schoolchildren (22.9%–28.1%) [47]. A possible explanation of the gap here described could be offered, besides age, gender, and ethnicity, by metabolic variables. This is in agreement with the statement that sdLDL increase is directly related to the number of components of the MetS mainly determined by TG concentrations [48].

SdLDL show increased susceptibility to oxidation, thus promoting endothelial damage and infiltrating the arterial wall [49]. These mechanisms activate foam cell growth, induce inflammation, and trigger a proatherosclerotic mechanism.

Moreover, sdLDL shows a strong correlation with the common cIMT, as outlined in a prospective study conducted in healthy males submitted to B-mode ultrasound, so providing evidence of cardiovascular risk by this surrogate marker [13].

Concerning the predictive usefulness of sdLDL on cardiovascular disease risk, results are conflicting. On the basis of multivariate analysis, and after doing adjustment for confounding variables, in particular TG and HDL cholesterol, this association was confirmed or not as an independent variable [43, 50, 51].

If the raised sdLDL particles relevance to coronary heart disease should be ascribed to their increased number or to the particle size “*per se*” is questionable [51]. Increased particle numbers might amplify the atherogenicity independently of the particle size and subjects with a predominance of sdLDL have significantly more particles than those with a predominance of larger, more buoyant LDL [49].

Thus, any final relationship between sdLDL and increased cardiovascular risk is not proven and the conclusion to consider LDL size a significant and independent predictor of cardiovascular risk is still debated.

In conclusion, according to ESC/EAS Guidelines, determination of sdLDL may be regarded as an emerging risk factor that may be used in the future but is not currently recommended for risk estimation [52].

### 3.1. Biochemical Markers of Atherogenic Disease

The cardiovascular risk prevention represents now a hot topic in children. Thus, several studies focus on identifying the link between unfavorable biomarkers profile and/or the presence of clinical indicators of atherosclerosis and the increased risk of cardiovascular disease [13, 53].

Biochemical markers of atherogenic particles, including LDL cholesterol, ApoB, and non-HDL cholesterol, are available and widely analyzed in both children and adults [53].

LDL cholesterol is considered the “gold standard” parameter for characterizing the cardiovascular disease risk when isolated hypercholesterolemia occurs, but its power decreases when TG levels are increased [53].

Non-HDL cholesterol includes TG-rich lipoproteins, remnants of TG-rich lipoproteins, and lipoprotein(a), so the non-HDL cholesterol values represent a better predictive indicator of cardiovascular disease than LDL cholesterol one, as demonstrated by observational and intervention studies [53]. Furthermore, non-HDL cholesterol correlates highly with plasma ApoB levels, now the best parameter to evaluate cardiovascular disease risk [53]. Both non-HDL cholesterol and ApoB usefulness have been recognized in predicting subclinical atherosclerosis by measuring cIMT [50, 54, 55].

According to the Expert Panel on Integrated Guidelines for Cardiovascular Health and Risk Reduction in Children and Adolescents [56], the evaluation of nonfasting non-HDL cholesterol among children and adolescents represents the first step to identify dislipidemia in pediatric age group and preventing the development of atherosclerosis. These recommendations [56] confirm that the first-line approach to primary prevention in children with dyslipidemia involves lifestyle modification. The use of pharmacologic treatment of lipid disorders in children is recommended in selective cases, due to the lack of long term safety and efficacy data [56, 57].

It has to be acknowledged that non-HDL cholesterol levels in children and adolescents vary with age, sex, and race/ethnicity [58]. Thus, it is important that own population reference values [5, 59] are used in the clinical practice to identify cardiovascular risk factors in pediatric age group.

The pathogenic linkage between obesity and metabolic and cardiovascular diseases leads to an increased attention to the relationship between adipocytokines levels and cardiovascular pathology [60, 61].

Among the identified adipokines related to cardiovascular disease [61], adiponectin represents an important player linking metabolic and cardiovascular alterations. Adiponectin represents a marker of MetS in childhood obesity [62] and has been associated with signs of cardiovascular disease in youths, as assessed by cIMT [63]. Furthermore, findings from the Young Finns study [64] showed a decreased 6-year incidence of MetS in young adults with low adiponectin levels and a strict association between adiponectin and high carotid IMT in subjects affected by MetS.

Moreover, Stakos et al. [60] have recently demonstrated a significant association between alterations in plasma leptin and adiponectin levels and high sensitivity C-reactive protein (hs-CRP), a well known biomarker of cardiovascular risk [53] in 170 nonobese children. Thus, these findings confirm the role of leptin and adiponectin as markers of cardiometabolic risk.

### 3.2. Subclinical Markers of Atherogenic Disease

A growing body of evidence supports the role of subclinical indicators of atherosclerosis such as increased cIMT assessed with ultrasound, increased left ventricular mass with cardiac ultrasound, endothelial dysfunction (reduced arterial dilation) with brachial ultrasound imaging, and the demonstration of coronary calcium on electron beam computed tomography imaging [56, 65, 66].

The association between cIMT and atherosclerosis biomarkers has been shown by several studies in adults and in children [56, 65, 67]. In adults, increased cIMT is associated with several cardiovascular risk factors including age, male sex, diabetes mellitus, total cholesterol, and smoking and it is also predictive of future cardiovascular events, including stroke and myocardial infarction [67, 68]. In children, there is an open debate in literature on the possible influence of age and obesity on the cIMT increase [13, 66, 69]. Notably, by analyzing 24 prepubertal children with a familial positive history of premature cardiovascular disease, de Giorgis et al. [70] ruled out the possible influence of blood pressure and age on cIMT. The authors showed a direct correlation between cIMT and oxidative stress marker, suggesting the use of ultrasound technique as reliable method to evaluate abnormalities in vascular wall early in life.

Brachial arterial flow-mediate dilatation (FMD) is further noninvasive method to evaluate functional changes in the arterial wall [13, 66]. Most of the studies reported lower FMD in obese than normal weight children [13, 66, 71]. Studies in children showed an inverse correlation between FMD and insulin resistance indexes [72] and a negative association of FMD with LDL cholesterol [62, 73].

Among the subclinical atherosclerosis markers in children, measurement of coronary artery calcifications (CAC) is emerging as important indices of future atherosclerosis [74]. In adult patients, electron-beam CT has a high level of sensitivity in detecting and defining the location and extent of CAC, which predicts obstructive coronary artery disease, and has prognostic value for future coronary events [75].

Wong et al. [75] demonstrated a significantly greater prevalence of CAC in both men and women with a self-reported history of hypertension and hypercholesterolemia. Also, a significant relationship of coronary risk factors measured during childhood and young adult life with the presence of CAC was noted [76]. Experience with these technologies in pediatric patients is limited to selected patients [77, 78].

Recently, Bacha et al. [79] have evaluated markers of subclinical atherosclerosis in ninety obese youth.

A total of 50% of patients showed CACs and adiposity as the major determinant of these vascular alterations [79].

Further research is needed to establish how cardiovascular risk factors affect clinical sign of cardiovascular disease.

## 4. Old and Novel Methods to Study Lipid Profile

Traditional analyses of lipids and FA include spectrophotometric kits to measure serum levels of TC and TG and high performance liquid chromatographic (HPLC) separation using normal-phase techniques and evaporative light-scattering detection enables the separation of major classes of lipids, such as cholesteryl esters, TG, free cholesterol, phosphatidylethanolamines, phosphatidylcholines, sphingomyelins, and lysophospholipids, as well as gas chromatography of FA composition of total serum phospholipids or cholesteryl esters, which is still a method of choice with respect to interpreting fatty acid metabolism. Other common lipid analyses methods include thin-layer chromatography (TLC), gas chromatography (GC), and nuclear magnetic resonance (NMR) spectroscopy.

However, the recent expansion in research in the field of lipidomics has been driven by the development of new tools and protocols for the identification and quantification of molecular lipids in different biological samples [80]. Mass spectrometry-based techniques occupy a leading position in the characterization, identification, and quantitation of lipids. They include ionization by electrospray (ESI), matrix-assisted laser desorption/ionization (MALDI), tandem mass spectrometry (MS/MS or MSn), fast atom bombardment (FAB), atmospheric pressure chemical-ionization (APCI), and atmospheric pressure photo-ionization (APPI). Selecting a particular MS-based method depends on the approach used (global or targeted) and on lipid class to be analyzed [81].

Data analysis can be performed with different approaches, even though the most successful are those megavariate statistics incorporating existing biological knowledge into the statistical analysis, enabling the generation of integrative knowledge for systems level investigation of lipidomics [82].

Interestingly, more recently the application of advanced integrated methodologies to analyze global lipidomics in human plasma has revealed a remarkable diversity of lipids that may represent an individual signature of the roles of lipids in health and diseases; thus it seems likely that plasma lipidome has emerged as one of the tool-sets for bringing it to practice.

On the bioinformatics side, the major limitation is the virtual absence of comprehensive and integrated reference databases. Communication of results from the global analysis of cellular lipidomes is complicated by the convoluted nomenclature of lipids. Lipid Analytical Tool (LIPIDAT) and Lipid Bank provide some information on structure and nomenclature but are not comprehensive enough on functional information and the provision of links to protein and gene data. Current efforts in both the public sector (e.g., LIPID Metabolites and Pathway Strategy) and private sector (e.g., lipidomics) are addressing this need. Databases and search algorithms will also aid in the annotation of known lipid metabolites and the identification of novel lipid metabolites. Future reference bases will be more integrated and take into account our knowledge of lipid biosynthetic pathways and protein-lipid interactions. The availability of such databases will be a prerequisite for future integration of lipidomics into proteomics and genomics.

## 5. Conclusions

In conclusion, a growing number of scientific data support that atherosclerotic process begins in childhood and represents an increasing health problem in obese children and adolescents. Visceral fat distribution and insulin resistance represent important markers of its progression. Further research is needed to identify early indicators of lipid alterations and to fully define the underlying pathophysiology. The complete characterization of the mechanisms involved in the development of atherogenic dyslipidemia in children could help in the identification of preventive and therapeutic strategies and thus leads to the reduction of the associated cardiovascular complications later in life.
